# Supplementary material for: Biomolecular condensates mediate bending and scission of endosome membranes
Source: Nature. 2024 Oct 9;634(8036):1204–10. doi: 10.1038/s41586-024-07990-0 (PMC11525194; doi:10.1038/s41586-024-07990-0)
Supplement: Supplementary file 1 — Supplementary Notes 1 and 2, Supplementary Figs. 2–4, Supplementary Table 1 and Supplementary References. [file 41586_2024_7990_MOESM1_ESM.pdf]

nature portfolio

https://doi.org/10.1038/s41586-024-07990-0

Supplementary information

Biomolecular condensates mediate bending and scission of endosome membranes

In the format provided by the authors and unedited

Supplementary Theory 1: Simulations of vesicle wetting dynamics

1. Numerical model

In this section, we present the numerical model used to simulate the wetting dynamics of biological membranes in contact with liquid condensates that result in the formation of ILVs within MVBs by FREE1 condensates. The model combines the ALE method to describe the surface motion [1] with the phase field method for an implicit description of the condensate(s) [2]. The combination of both methods has been introduced in Ref. [3] to describe condensates in contact with a viscoelastic solid material. Here we extended the work in [3] to include elastic surface forces of the membrane. These forces are coupled monolithically to the bulk equations for fluid flow inside and outside the surface as detailed in the following.

1.1. Configuration

We consider a fluid domain $\Omega \in \mathrm{R}^d$, which is separated by a membrane Γ into two subdomains $\Omega_{\beta\gamma}$ and Ω_α, which denote the regions outside and inside of Γ, respectively. An illustration is given in Fig. 2.The membrane is assumed to be infinitely thin. Outside the membrane, i.e. in $\Omega_{\beta\gamma}$, a two-phase fluid is considered, one phase describing the condensate (subscript β), the other one describing a surrounding ambient fluid phase (subscript γ). In order to indicate the fluid phases, an order parameter ϕ is introduced, referred to as phase field, with $\phi = 0$ in the ambient phase and $\phi = 1$ in the condensate phase. Across the ambient-liquid interface, the phase field varies smoothly, following a tangent hyperbolic profile. This leads to a thin diffuse interface of width ε [4]. Three surface tensions are present along the three contact lines. A fluid-fluid tension $\sigma_{\beta\gamma}$ contracts the interface described by the phase-field and two membrane-fluid tensions, $\sigma_{\alpha\gamma}$ and $\sigma_{\alpha\beta}$, contract the membrane surface which is in contact with the ambient and condensate phase, respectively. The membrane, in addition to its surface tension, reacts to out-of-plane deformations with a bending stiffness force. In-plane deformations are penalized with a stretching force based on the stretching elasticity of the membrane material.

1.2. Governing equations

1.2.1. Fluid equations

We use the phase field model from [5] which considers a volume-averaged velocity formulation with fluid velocity $\mathbf{v}$ and reads

$$\left.\begin{aligned} \rho(\phi)\left(\partial_t \mathbf{v}_i + \mathbf{v}_i \cdot \nabla \mathbf{v}_i\right) - \nabla \cdot \mathbf{S}_i &= \mathbf{0} \\ \nabla \cdot \mathbf{v}_i &= 0 \end{aligned}\right\} \text{ in } \Omega_i, i \in \{\alpha, \beta\gamma\} \tag{1}$$

$$\left.\begin{aligned} \partial_t \phi + \mathbf{v}_{\beta\gamma} \cdot \nabla \phi &= \nabla \cdot (m \nabla \mu) \\ \mu &= \frac{\tilde{\sigma}}{\varepsilon} W'(\phi) - \tilde{\sigma}\varepsilon \Delta \phi \end{aligned}\right\} \text{ in } \Omega_{\beta\gamma} \tag{2}$$

with viscous pressure and capillary stress

$$\mathbf{S}_i = \eta(\phi)\left(\nabla \mathbf{v}_i + \nabla \mathbf{v}_i^T\right) - p_i \mathbf{I} - \underbrace{\tilde{\sigma}\varepsilon \nabla \phi \otimes \nabla \phi}_{\mathbf{S}_{ca}}, \tag{3}$$

where $\phi = 0$ in Ω_α. Here, the following notations were used: the subscript $i \in \{\alpha, \beta\gamma\}$, referring to the respective subdomain, the phase-dependent fluid density ρ, constant mobility m,

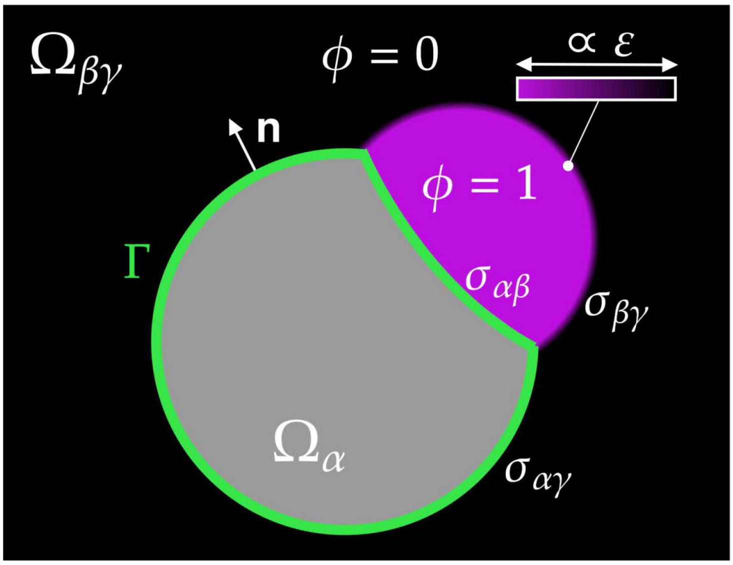

Figure 2: Illustration of the FREE1 condensate wetting the MVB membrane scenario. A deformable closed membrane Γ encloses a fluid domain Ω_α and borders on a two-phase fluid domain $\Omega_{\beta\gamma}$. The two fluids in $\Omega_{\beta\gamma}$ are indicated by the value of the phase field function ϕ. The fluid-fluid interface is diffuse with a thickness ε and carries a surface tension $\sigma_{\beta\gamma}$. The membrane Γ has two distinct surface tensions $\sigma_{\alpha\gamma}$ and $\sigma_{\alpha\beta}$ depending on the contacting fluid.

the chemical potential μ, the double well potential W, the (scaled) fluid-fluid surface tension $\tilde{\sigma}$, and the phase-dependent viscosity η. In our case, the double well potential is chosen as $W(\phi) = \frac{1}{4}\phi^2(1-\phi)^2$, which yields a scaling of the physical surface tension $\sigma_{\beta\gamma}$ according to $\tilde{\sigma} = 6\sqrt{2}\sigma_{\beta\gamma}$, see, for example, Reference [5].

1.2.2. Coupling conditions

The following coupling conditions apply at the interface Γ

$$\left.\begin{aligned} \mathbf{v}_{\beta\gamma} &= \mathbf{v}_\alpha \\ \mathbf{S}_{\beta\gamma}\mathbf{n} &= \mathbf{S}_\alpha\mathbf{n} - \nabla_\Gamma \cdot (\sigma_s(\phi)\mathbf{P}) - \frac{\partial E_{\text{bend}}}{\partial \Gamma} - \frac{\partial E_{\text{stretch}}}{\partial \Gamma} \\ \tilde{\sigma}\varepsilon\mathbf{n} \cdot \nabla\phi &= -\sigma_s'(\phi) \\ \mathbf{n} \cdot \nabla\mu &= 0 \end{aligned}\right\} \quad \text{on } \Gamma. \tag{4}$$

The first equation is the continuity of velocities across the interface which is employed due to the assumption of an infinitely thin membrane Γ. The second equation is the dynamic condition that describes the balance of forces across the interface (namely traction, capillary stress, bending stiffness, and stretching elasticity). Here, $\mathbf{n}$ is the outer normal to Ω_α. The surface tension $\sigma_s(\phi)$ represents the tension along Γ between the membrane and the fluid indicated by the value of the phase field. We use the following differentiable function of the phase field

$$\sigma_s(\phi) = (\sigma_{\alpha\beta} - \sigma_{\alpha\gamma})\phi^2(3 - 2\phi) + \sigma_{\alpha\gamma}, \tag{5}$$

which implies $\sigma_s(0) = \sigma_{\alpha\gamma}$ and $\sigma_s(1) = \sigma_{\alpha\beta}$. In addition, $\nabla_\Gamma \cdot$ denotes the surface divergence operator and $\mathbf{P} = \mathbf{I} - \mathbf{n} \otimes \mathbf{n}$ the surface projection operator. Note that the present formulation coincides with the boundary conditions given in References [6], [7] and [8] as $\nabla_\Gamma \cdot (\sigma_s(\phi)\mathbf{P}) = 2\sigma_s(\phi)M\mathbf{n} + \nabla_\Gamma\sigma_s(\phi)$ with $M = -\frac{1}{2}\nabla_\Gamma \cdot \mathbf{n}$ being the mean curvature of the membrane.

The bending force is described by the first variation of the bending energy

$$E_{\text{bend}} = 2c_b \int_\Gamma M^2 \text{d}A \tag{6}$$

with the material specific bending stiffness c_b $[Nm]$. The stretching energy E_{stretch} minimizes in-plane stretching and compression of the membrane compared to the reference state. Several formulas for the membrane stretching energy have been proposed in the literature (e.g. [9, 10]), leading to slightly different formulas for the resulting stretching force. Here, we use the stretching energy [11]

$$E_{\text{stretch}} = \int_\Gamma \frac{K_A}{8} \text{tr}^2 (\mathbf{B} - \mathbf{P}) \, \text{dA} \tag{7}$$

with the left Cauchy-Green strain tensor $\mathbf{B}$ and the area dilation modulus K_A [11], respectively.

The third equation, Eq. $(4)_3$, is responsible for the formation of a static contact angle.

Finally, Eq. $(4)_4$ ensures mass conservation (no penetration) on Γ. With this, we have specified a closed system of equations (up to outer boundary conditions) for the unknown variables $\mathbf{v}_i, p_i, \phi$, and μ $(i \in \{\alpha, \beta\gamma\})$.

1.2.3. Discretization

The system is discretized using the finite element toolbox AMDiS [12]. An IMEX (implicit/explicit) Euler method is used to formulate a time discretization of Equations Eq. (1)-Eq. (4) which is linear in the solution variables. The surface is described with a surface grid that is moved in time with the ALE method [1]. In order to compute the bending stiffness force on the surface, a scheme similar to that in ([13], Sec. 9) is used. The computation of the stretching force is described in more detail in [1]. The resulting surface equations are solved monolithically together with the bulk equations Eq. (1)-Eq. (3).

1.2.4. Simulation setup and parameters

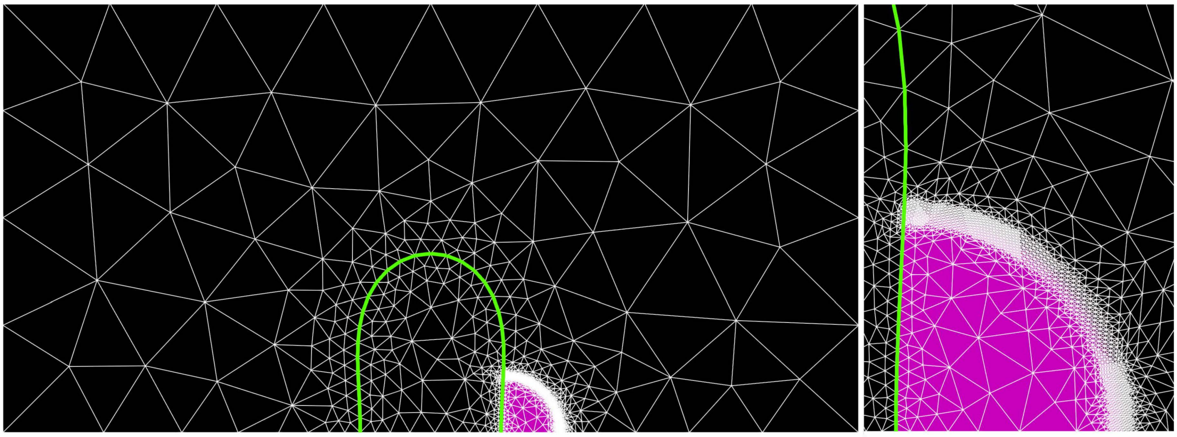

Figure 3: Mesh of the GUV simulations in the initial state. **left:** full view, **right:** close up view at the condensate. The center of the membrane (green) is located at the origin of the coordinate system. The condensate is placed on the right and is initially a half sphere. The bottom boundary of the mesh is the symmetry axis.

The simulations in this work have been performed in 3D axisymmetric. The stretching force is chosen to be sufficiently high, so that the membrane extension locally is less than 1%, that

is, the membrane is nearly inextensible. The surface tensions $\sigma_{\beta\gamma}$, $\sigma_{\alpha\beta}$ and $\sigma_{\alpha\gamma}$ are chosen in a way such that for a flat surface, the contact angle θ of the condensate fulfils Young's relation

$$\cos\theta = \frac{\sigma_{\alpha\gamma} - \sigma_{\alpha\beta}}{\sigma_{\beta\gamma}}, \tag{8}$$

and $\theta = 70°$.

The domain in the GUV simulations (see Fig. 2) is rectangular with a width of 30μm and a height of 15μm. The bottom boundary of the mesh is located on the $x-$ axis and therefore is the symmetry axis. The initial membrane shape is prescribed by a minimum energy configuration, which minimizes the bending energy under the constraints of fixed are and volume. This shape is created by running a preliminary simulation (without condensate) of an incompressible membrane with bending stiffness. The preliminary simulation is initialized with a cigar-shaped membrane (i.e. a shape composed of a cylinder with half-spheres on the top and bottom) created such that there is exactly enough membrane excess area to fully enclose the droplet. More precisely, we assume that the resulting volume of the surface of revolution is equal to the volume of a sphere with radius $r = 5\mu$m, which has a spherical hole inside. This hole has the volume of the droplet. The resulting area is made up of the area of a sphere of radius $r = 5\mu$m plus the area of the condensate in its spherical state. Given these two information, and assuming that the condensate in its spherical state has a radius of r_d, one can compute the distance $2d$ between the two parallel lines of the cigar and the length d of these lines solving the following system:

$$2\pi\left(h^2 + \pi d\left(h + \frac{2d}{\pi}\right)\right) = 4\pi\left(r^2 + r_d^2\right)c \tag{9}$$

$$2\pi d\left(h^2 + \left(h + \frac{4d}{3\pi}\right)\frac{\pi d}{2}\right) = \frac{4}{3}\pi\left(r^3 - r_d^3\right), \tag{10}$$

where the left hand side of Eq. (9) and Eq. (10) describe the area and the volume of the cigar shaped surface of revolution. Note that this surface of revolution is basically the outer half of a torus of major radius h and minor radius d, attached to a cylinder of radius d and height h. Here, $c = 1.03$ is chosen, which ensures that the vesicle has enough excess area to engulf the condensate even if the bending force is large.

The resulting membrane has an area of $A = 355.33\mu\text{m}^2$ and a volume of $V = 504.15\mu\text{m}^3$, which are both constant in time due to fluid and membrane incompressibility. The condensate is initially placed as a half spherical cap of radius 2μm at the center on the right side of the vesicle. Hence, $r_d = 0.5^{\frac{1}{3}} \cdot 2 = 1.59\mu$m and the volume of the condensate in the initial state is $V_d = 16.76\mu\text{m}^3$.

Finally, the following boundary conditions were imposed on the outer boundary of the computational domain:

$$\left.\begin{aligned} \mathbf{v}_{\beta\gamma} &= 0 \\ \mathbf{m}\cdot\nabla\phi &= 0 \\ \mathbf{m}\cdot\nabla\mu &= 0 \end{aligned}\right\} \text{on } \partial\Omega \tag{11}$$

with the unit outer normal $\mathbf{m}$ to $\partial\Omega$. Furthermore, to obtain a unique solution for the pressure, $p = 0$ holds on the left and right boundary of Ω.

References

[1] M. Mokbel, S. Aland, An ale method for simulations of axisymmetric elastic surfaces in flow, International Journal for Numerical Methods in Fluids 92 (11) (2020) 1604–1625.

[2] S. Aland, A. Voigt, Benchmark computations of diffuse interface models for two-dimensional bubble dynamics, International Journal for Numerical Methods in Fluids 69 (3) (2012) 747–761.

[3] S. Aland, D. Mokbel, A unified numerical model for wetting of soft substrates, International Journal for Numerical Methods in Engineering 122 (4) (2021) 903–918.

[4] D. Jacqmin, Contact-line dynamics of a diffuse fluid interface, Journal of Fluid Mechanics 402 (2000) 57–88.

[5] H. Ding, P. D. Spelt, C. Shu, Diffuse interface model for incompressible two-phase flows with large density ratios, Journal of Computational Physics 226 (2) (2007) 2078–2095.

[6] E. H. van Brummelen, M. S. Roudbari, G. Şimşek, K. G. van der Zee, 8. Binary-fluid–solid interaction based on the Navier–Stokes–Cahn–Hilliard Equations, De Gruyter, 2017, Ch. 8, pp. 283–328.

[7] E. H. van Brummelen, M. Shokrpour-Roudbari, G. J. van Zwieten, Elasto-Capillarity Simulations Based on the Navier–Stokes–Cahn–Hilliard Equations, Springer International Publishing, 2016, Ch. 0, pp. 451–462.

[8] H. Van Brummelen, T. Demont, G. Van Zwieten, An adaptive isogeometric analysis approach to elasto-capillary fluid-solid interaction, International Journal for Numerical Methods in Engineering 122.

[9] C. Pozrikidis, Numerical simulation of the flow-induced deformation of red blood cells, Annals of Biomedical Engineering 31 (10) (2003) 1194–1205.

[10] D. Le, J. White, J. Peraire, K. Lim, B. Khoo, An implicit immersed boundary method for three-dimensional fluid–membrane interactions, Journal of Computational Physics 228 (22) (2009) 8427 – 8445.

[11] M. Mokbel, An ale method for simulations of elastic surfaces in flow, dissertation, Technische Universität Bergakademie Freiberg (2021).

[12] S. Praetorius, The Adaptive Multi-Dimensional simulation toolbox (AMDiS), a discretization module on top of the Dune framework., `https://gitlab.com/amdis/amdis`.

[13] J. W. Barrett, H. Garcke, R. Nürnberg, Parametric finite element approximations of curvature driven interface evolutions, Vol. 21, 2020, pp. 275–423. `doi:10.1016/bs.hna.2019.05.002`.
URL `https://arxiv.org/pdf/1903.09462.pdf`

Supplementary Theory 2: Droplet-induced neck constriction forces

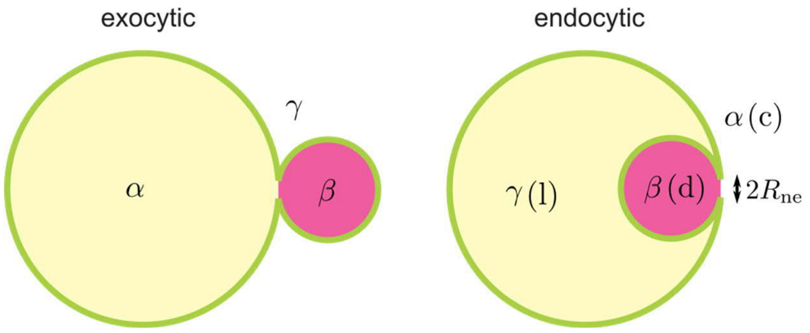

Fig.4: Geometry of the final stage of droplet engulfment by a membrane. Left: "exocytic" case with the droplet wetting from the inside. Right: "endocytic" case with the droplet wetting from the outside. The engulfment of FREE1 condensates by MVB membranes is endocytic, where the phases $\gamma, \alpha,$ and β can be identified with vacuole lumen (l), cytosol (c), and droplet (d) respectively. The radius of the neck is given by R_{ne}.

2.1. General results for exocytic and endocytic droplet engulfment

We study configurations such as those depicted in Fig. 3, corresponding to "exocytic" (left) and "endocytic" (right) engulfment of a droplet by a vesicle. This model is applicable to a diversity of droplets including biomolecular condensates in cells, coacervates or polymeric phases. Here, we are interested in particular in configurations of endocytic type, as they resemble the invagination and scission of ILVs from MVB membranes by FREE1 condensates (main Figs. 3c, 4b, 5d).

In order to understand when configurations with very narrow, "closed" membrane necks are stable, we need to consider how the energy of the system depends on the radius of the neck R_{ne}. This energy $E(R_{\mathrm{ne}})$ will include contributions corresponding to both the bending energy of the membrane, the wetting and surface tension energies of the droplet, and the line tension of the three-phase contact line. A scaling analysis (Lipowsky, 2018) shows that the droplet interfacial tension only contributes to order $O(R_{\mathrm{ne}}^2)$, whereas the bending and line tension contributions do so to order $O(R_{\mathrm{ne}})$.

Nevertheless, the presence of the droplet still has a strong effect, as it affects the spontaneous curvature and bending rigidity of the membrane segment in contact with it, and generates the line tension. It can be calculated (Jülicher & Lipowsky, 1996; Lipowsky, 2018) that the energy $\Delta E(R_{\mathrm{ne}}) \equiv E(R_{\mathrm{ne}}) - E(R_{\mathrm{ne}} = 0)$ goes as

$$\Delta E(R_{\mathrm{ne}}) = 4\pi R_{\mathrm{ne}}\left[-\kappa_{\alpha\gamma}\left(M_{\alpha\gamma} - m_{\alpha\gamma}\right) - \kappa_{\beta\gamma}\left(M_{\beta\gamma} - m_{\beta\gamma}\right) + \lambda/2\right] + O(R_{\mathrm{ne}}^2) \qquad (1)$$

in the exocytic case ($M_{\alpha\gamma} > 0$ and $M_{\beta\gamma} > 0$); and as (Agudo-Canalejo & Lipowsky, 2016)

$$\Delta E(R_{\mathrm{ne}}) = 4\pi R_{\mathrm{ne}}\left[\kappa_{\gamma\alpha}\left(M_{\gamma\alpha} - m_{\gamma\alpha}\right) + \kappa_{\gamma\beta}\left(M_{\gamma\beta} - m_{\gamma\beta}\right) + \lambda/2\right] + O(R_{\mathrm{ne}}^2) \qquad (2)$$

in the endocytic case ($M_{\gamma\alpha}$ > 0 and $M_{\gamma\beta}$ < 0). Here, each membrane segment has its corresponding bending rigidity κ_{ij}, spontaneous curvature m_{ij}, and mean curvature M_{ij}, and the line tension of the three-phase contact line is given by λ.

From (1) and (2), we deduce that the force $f = \left.\frac{\mathrm{d}E(R_{\mathrm{ne}})}{\mathrm{d}R_{\mathrm{ne}}}\right|_{R_{\mathrm{ne}}=0}$ exerted at the neck (Agudo-Canalejo & Lipowsky, 2016) is given by

$$f = -4\pi\left[\kappa_{\alpha\gamma}\left(M_{\alpha\gamma} - m_{\alpha\gamma}\right) + \kappa_{\beta\gamma}\left(M_{\beta\gamma} - m_{\beta\gamma}\right)\right] + 2\pi\lambda \qquad (3)$$

in the exocytic case ($M_{\alpha\gamma}$ > 0 and $M_{\beta\gamma}$ > 0); and by

$$f = 4\pi\left[\kappa_{\gamma\alpha}\left(M_{\gamma\alpha} - m_{\gamma\alpha}\right) + \kappa_{\gamma\beta}\left(M_{\gamma\beta} - m_{\gamma\beta}\right)\right] + 2\pi\lambda \qquad (4)$$

in the endocytic case ($M_{\gamma\alpha}$ > 0 and $M_{\gamma\beta}$ < 0). Here, we use the notation that the force is positive f > 0 when it is constrictive.

In order for a closed neck to be stable, we need $f \geq 0$. This results in the conditions for closure of the neck

$$\lambda/2 + \kappa_{\alpha\gamma}m_{\alpha\gamma} + \kappa_{\beta\gamma}m_{\beta\gamma} \geq \kappa_{\alpha\gamma}M_{\alpha\gamma} + \kappa_{\beta\gamma}M_{\beta\gamma} \qquad (5)$$

in the exocytic case ($M_{\alpha\gamma}$ > 0 and $M_{\beta\gamma}$ > 0); and

$$\lambda/2 - \kappa_{\gamma\alpha}m_{\gamma\alpha} - \kappa_{\gamma\beta}m_{\gamma\beta} \geq -\kappa_{\gamma\alpha}M_{\gamma\alpha} - \kappa_{\gamma\beta}M_{\gamma\beta} \qquad (6)$$

in the endocytic case ($M_{\gamma\alpha}$ > 0 and $M_{\gamma\beta}$ < 0). If these conditions are not satisfied, droplet engulfment may still be possible, but there will be a finite-sized, wide-open neck. The inequalities (5) and (6) show that neck closure is always favored by a positive line tension λ > 0, and moreover by positive spontaneous curvatures in the exocytic case and negative spontaneous curvatures in the endocytic case.

The existence of membrane-mediated forces at the neck that will promote membrane fission was first predicted in the context of the engulfment of solid particles by membranes in (Agudo-Canalejo & Lipowsky, 2016), further explored in the context of membrane fission by ESCRT proteins in (Agudo-Canalejo & Lipowsky, 2018), and lastly confirmed experimentally in (Steinkühler et al., 2020), for the case of budding of uniform membranes induced by spontaneous curvature, which coincides with the limit of negligible adhesion of the original result in (Agudo-Canalejo & Lipowsky, 2016). The experimental measurements of Steinkühler et al. showed that forces at the neck above a critical value $f_* \approx 25$ pN were sufficient to cause membrane fission.

2.2. Invagination and scission of MVB membranes by FREE1 condensates

FREE1 condensate mediated invaginations correspond to the endocytic case where we identify phase γ as the vacuole lumen (l), phase α as the cytosol (c), and phase β as the droplet (d). The radius R_{v} of the vacuole determines the mean curvature $M_{\mathrm{lc}} = 1/R_{\mathrm{v}}$, and the radius of the droplet determines the mean curvature $M_{\mathrm{ld}} = -1/R_{\mathrm{d}}$. With these substitutions, the condition for closed necks (Eq. 6) becomes

$$\lambda/2 - \kappa_{\mathrm{lc}} m_{\mathrm{lc}} - \kappa_{\mathrm{ld}} m_{\mathrm{ld}} \geq \kappa_{\mathrm{ld}}/R_{\mathrm{d}} - \kappa_{\mathrm{lc}}/R_{\mathrm{v}} \qquad (7)$$

and the force exerted at the neck is given by

$$f = 4\pi[\kappa_{\mathrm{lc}}(1/R_{\mathrm{v}} - m_{\mathrm{lc}}) - \kappa_{\mathrm{ld}}(1/R_{\mathrm{d}} + m_{\mathrm{ld}})] + 2\pi\lambda. \qquad (8)$$

For simplicity, we may assume that κ_{lc} and κ_{ld} are of the same order of magnitude, and that the lc-segment does not have a strong spontaneous curvature. With these two assumptions, the expression for the force becomes

$$f \approx -4\pi\kappa_{\mathrm{ld}}(1/R_{\mathrm{d}} - 1/R_{\mathrm{v}} + m_{\mathrm{ld}}) + 2\pi\lambda. \qquad (9)$$

A positive constriction force therefore requires either sufficiently negative m_{ld} and/or sufficiently positive λ. Let us consider two limiting cases: (i) the spontaneous curvature dominates and (ii) the line tension dominates.

For dominating spontaneous curvature (negligible line tension), the condition for fission $f > f_*$ becomes

$$m_{\mathrm{ld}} < -\left(\frac{1}{R_{\mathrm{d}}} - \frac{1}{R_{\mathrm{v}}} + \frac{f_*}{4\pi\kappa_{\mathrm{ld}}}\right). \qquad (10)$$

Using typical values of the FREE1 condensate and MVB radii $R_{\mathrm{d}} = 17.5$ nm and $R_{\mathrm{v}} = 100$ nm respectively, typical value for the bending rigidity $\kappa_{\mathrm{ld}} = 20 k_{\mathrm{B}}T \approx 80$ pN · nm, and the experimentally measured critical neck constriction force $f_* = 25$ pN (Steinkühler et al., 2020), we obtain $m_{\mathrm{ld}} < -1/(14\text{ nm})$. The corresponding condition for neck closure without fission (given by (10) with $f_* = 0$) is $m_{\mathrm{ld}} < -1/(21\text{ nm})$. While these values of spontaneous curvature seem rather high, they are within the range of spontaneous curvatures generated not only by specialized curvature-inducing proteins, but also by generic His-tagged proteins such as GFP binding to NTA-lipids in the membrane (Steinkühler et al., 2020).

For dominating line tension (negligible spontaneous curvature), we obtain the condition for fission

$$\lambda > 2\kappa_{\mathrm{ld}}\left(\frac{1}{R_{\mathrm{d}}} - \frac{1}{R_{\mathrm{v}}}\right) + \frac{f_*}{2\pi}. \qquad (11)$$

Using the same values of R_{d}, κ_{ld}, and f_* as above, we obtain $\lambda > 11.5$ pN. The corresponding condition for neck closure without fission (given by (11) with $f_* = 0$) is $\lambda > 7.5$ pN. Line tensions of the order of $1 - 100$ pN are typical for three-phase contact lines, as calculated from theory and measured in simulations and experiments for a wide range of non-biological systems (reviewed in Law et al., 2017). Experimental measurements of line tension between biomolecular condensates and lipid membranes do not exist at the moment. However, dissipative particle dynamics (DPD) simulations of the engulfment of nano-sized droplets by lipid vesicles measured line tensions at the three-phase contact line which, depending on the composition of the membrane, could have positive and negative values ranging from -130 pN to +100 pN (Ghosh et al., 2023). Consistently with our results, spontaneous fission of the neck was observed in these DPD simulations only when the line tension was positive.

2.3. Neck closure and fission mediated purely by bending rigidity contrast

Inspection of eq. (4) shows that even in the absence of spontaneous curvature or line tension, positive constriction forces may be obtained in the endocytic case if there is a sufficiently strong bending rigidity contrast. Indeed, the equation in this case reads:

$$f = 4\pi\left[\kappa_{\gamma\alpha} M_{\gamma\alpha} + \kappa_{\gamma\beta} M_{\gamma\beta}\right] \qquad (12)$$

where we remind that $M_{\gamma\alpha} > 0$ and $M_{\gamma\beta} < 0$ in the endocytic case. In particular, a stable closed neck ($f > 0$) can be obtained if

$$\frac{\kappa_{\gamma\alpha}}{\kappa_{\gamma\beta}} > \frac{|M_{\gamma\beta}|}{M_{\gamma\alpha}} \qquad (13)$$

In the example of droplet engulfment by vacuoles, this would correspond to

$$\frac{\kappa_{\mathrm{lc}}}{\kappa_{\mathrm{ld}}} > \frac{R_{\mathrm{v}}}{R_{\mathrm{d}}} \qquad (14)$$

These inequalities imply that a closed neck can be stable even in the absence of spontaneous curvature or line tension if contact with the droplet softens the membrane (decreases its bending rigidity) to a sufficient degree. This is however only possible in the endocytic case.

References for Supplementary Theory 2:

Agudo-Canalejo, J., & Lipowsky, R. (2018). Domes and cones: Adhesion-induced fission of membranes by ESCRT proteins. *PLoS Computational Biology*, *14*, e1006422.

Agudo-Canalejo, J., & Lipowsky, R. (2016). Stabilization of membrane necks by adhesive particles, substrate surfaces, and constriction forces. *Soft Matter*, *12*, 8155-8166.

Ghosh, R., Satarifard, V., & Lipowsky, R. (2023). Different pathways for engulfment and endocytosis of liquid droplets by nanovesicles. *Nature Communications*, 14, 615.

Law, B. M., McBride, S. P., Wang, J. Y., Wi, H. S., Paneru, G., Betelu, S., ... & Aratono, M. (2017). Line tension and its influence on droplets and particles at surfaces. *Progress in Surface Science*, *92*, 1-39.

Lipowsky, R. (2018). Response of membranes and vesicles to capillary forces arising from aqueous two-phase systems and water-in-water droplets. *The Journal of Physical Chemistry B*, *122*, 3572-3586.

Jülicher, F., & Lipowsky, R. (1996). Shape transformations of vesicles with intramembrane domains. *Physical Review E*, *53*, 2670.

Kusumaatmaja, H., & Lipowsky, R. (2011). Droplet-induced budding transitions of membranes. *Soft Matter*, *7*, 6914-6919.

Steinkühler, J., Knorr, R. L., Zhao, Z., Bhatia, T., Bartelt, S. M., Wegner, S., ... & Lipowsky, R. (2020). Controlled division of cell-sized vesicles by low densities of membrane-bound proteins. *Nature Sommunications*, *11*, 905.

Supplementary Table 1. Primers for constructs

Primer name	Prime sequence (5'-3')	Purpose
MBP-GFP-FREE1-F	GTGGTAGCGGCGGCTCCATGGGCCAACAGGGAGATTACAATTC	for recombinant MBP-GFP-FREE1
MBP-GFP-FREE1-R	ATGGTGATGGTGATGGTCGACATGTGCGCTAACGAGGAAAG	
MBP-GFP-FREE1$^{\Delta IDR}$-F	GTGGTAGCGGCGGCTCCATGGAATCAGTGAAATTTGATCAATC	for recombinant MBP-GFP-FREE1$^{\Delta IDR}$
MBP-GFP-IDR^{FUS} FREE1-F	GTGGTAGCGGCGGCTCCATGGAAGCCTCAAACGATTATACCCAAC	for recombinant MBP-GFP-IDR^{FUS}-FREE1
FREE1-IDR^{FUS}-R	GATTGATCAAATTTCACTGAGTCCTGCTGTCCATAGCCAC	
MBP-GFP-PTAP-IDR^{FUS}-FREE1-F	GTGGTAGCGGCGGCTCCATGGAACCTTCTCCGCCGGCGCCG	for recombinant MBP-GFP-PTAP-IDR^{FUS}-FREE1
MBP-GFP-IDR^{FUSm} FREE1-F	GTGGTAGCGGCGGCTCCATGGAAGCCTCAAACGATTCTACCC	for recombinant MBP-GFP-IDR^{FUSm}-FREE1
FUSm-R	GATTGATCAAATTTCACTGAGTCCTGCTGTCCAGAGCCAC	
MBP-GFP-IDR^{FLOE1} FREE1-F	CGCGGCAGCCATATGGCTAGCCACCAAATAGCCCCACAGCC	for recombinant MBP-GFP-IDR^{FLOE1}-FREE1
FLOE1PRLD-R	GATTGATCAAATTTCACTGAACCAATGTTACCGCCACCTG	
MBP-GFP-FREE1-R	CTTGCTCACTTTCATGGATCCATGTGCGCTAACGAGGAAAG	
MBP-FREE1-F	GTGCCGCGCGGCAGCCATATGATGCAACAGGGAGATTACAATTC	for recombinant MBP-FREE1
MBP-FREE1-R	GTGGTGGTGGTGGTGCTCGAGATGTGCG	

	CTAACGAGGAAAG	
MBP-FREE1$^{\Delta IDR}$-F	GTGCCGCGCGGCAGCCATATGTCAGTGA AATTTGATCAATC	for recombinant MBP-FREE1$^{\Delta IDR}$
mCherry-VPS23A-F<br>mCherry-VPS23A-R	GTGGTAGCGGCGGCTCCATGGGCGTTCC CCCGCCGTCTAATCC<br>GGTTTCTTTACCAGACTCGAGTCATGAAT GTAACCTACCTG	for recombinant VPS23-mCherry
mCherry-VPS37A-F<br>mCherry-VPS37A-R	GTGGTAGCGGCGGCTCCATGGGCTTCAA TTTCTGGGGATCAAAAG<br>GGTTTCTTTACCAGACTCGAGTCAAATGTT TGACGTTTTAGC	for recombinant VPS37-mCherry
mCherry-VPS28A-F<br>mCherry-VPS28A-R	GTGGTAGCGGCGGCTCCATGGGCGAGGT CAAGTTATGGAATG<br>GGTTTCTTTACCAGACTCGAGTTAATTACC AGCATTAGGC	for recombinant VPS28-mCherry
MBP-VPS23A-F<br>MBP-VPS23A-R	CTCTACTTCCAATCCCATATGGTTCCCCCG CCGTCTAATC<br>ATGGTGATGGTGATGGTCGACTGAATGTA ACCTACCTGCG	for recombinant MBP-VPS23
MBP-VPS37A-F<br>MBP-VPS37A-R	CTCTACTTCCAATCCCATATGTTCAATTTCT GGGGATCAAAAG<br>ATGGTGATGGTGATGGTCGACAATGTTTG ACGTTTTAGCGG	for recombinant MBP-VPS37
TOL6-Nco1-F<br>TOL6-Xho1-R	GTGGTAGCGGCGGCTCCATGGGCGCGTC GTCTTCAGCTTCGGC<br>GGTTTCTTTACCAGACTCGAGTTAAATCAT TTTCCTTCCTC	for recombinant TOL6-mCherry
TOL9-Nco1-F	GTGGTAGCGGCGGCTCCATGGGCGTGAA CGCTATGGTGGAGAG	for recombinant TOL9-mCherry

TOL9-Xho1-R	GGTTTCTTTACCAGACTCGAGTCACATGG TACCAGCTCTTC	
BRO1-Nco1-F	GTGGTAGCGGCGGCTCCATGGGCGCTTC TTCTTCGCTCTCTAATC	for recombinant BRO1-mCherry
BRO1-Xho1-R	GGTTTCTTTACCAGACTCGAGTCATTGCC TGTAGTATCCTC	
LIP5-Nco1-F	GTGGTAGCGGCGGCTCCATGGTCGAACC CAAACGAACCAGC	for recombinant LIP5-mCherry
LIP5-Xho1-R	GGTTTCTTTACCAGACTCGAGTCAGTGAC CGGCACCGGCCG	
VPS23A-FLAG-F	ATCTATCTCTCTCGAGGTACCATGGTTCCC CCGCCGTCTAATC	VPS23-FLAG for transient expression in *N.benthamiana*
VPS23A-FLAG-R	GGATGCGGCAGCAGAGGTACCTGAATGTA ACCTACCTGCG	
VPS28A-FLAG-F	ATCTATCTCTCTCGAGGTACCATGGAGGT CAAGTTATGGAATG	VPS28-FLAG for transient expression in *N.benthamiana*
VPS28A-FLAG-R	GGATGCGGCAGCAGAGGTACCATTACCA GCATTAGGCAAAG	
VPS37A-FLAG-F	ATCTATCTCTCTCGAGGTACCATGTTCAAT TTCTGGGGATC	VPS37-FLAG for transient expression in *N.benthamiana*
VPS37A-FLAG-R	GGATGCGGCAGCAGAGGTACCAATGTTTG ACGTTTTAGCGG	
TOL6-FLAG-F	ATCTATCTCTCTCGAGGTACCATGGCGTC GTCTTCAGCTTC	TOL6-FLAG for transient expression in *N.benthamiana*
TOL6-FLAG-R	GGATGCGGCAGCAGAGGTACCAATCATTT TCCTTCCTCCTATC	
TOL9-FLAG-F	ATCTATCTCTCTCGAGGTACCATGGTGAAC GCTATGGTGGAG	TOL9-FLAG for transient expression in *N.benthamiana*
TOL9-FLAG-R	GGATGCGGCAGCAGAGGTACCCATGGTA	

	CCAGCTCTTCCGG	
LIP5-FLAG-F	ATCTATCTCTCTCGAGGTACCATGTCGAACCCAAACGAACC	LIP5-FLAG for transient expression in *N.benthamiana*
LIP5-FLAG-R	GGATGCGGCAGCAGAGGTACCGTGACCGGCACCGGCCGATG	
BRO1-FLAG-F	ATCTATCTCTCTCGAGGTACCATGGCTTCTTCTTCGCTCTC	BRO1-FLAG for transient expression in *N.benthamiana*
BRO1-FLAG-R	GGATGCGGCAGCAGAGGTACCTTGCCTGTAGTATCCTCCAC	
FREE1pro-F	ACGACGGCCAGTGCCAAGCTTGCCCAGTTCCTCGCATACAC	*pFREE1::GFP-FREE1* transgenic construct
FREE1pro-R	GTTCTTCTCCTTTACTCATGTCGACCGCTGAATCGGTGATCGTCG	
GFP-Linker-F(P4)	ATCACCGATTCAGCGGAGCTCATGAGTAAAGGAGAAGAACTTTTC	
GFP-Linker-R(P5)	CCGGCGCGCCCACCCTTAGCGGTACCAAGCTCATCATGTTTGTATAG	
BamH1-FREE1-F	GGCCTGGACAGCACCGGATCCCAACAGGGAGATTACAATTC	
Sac1-FREE1-R	CGATCGGGGAAATTCGAGCTCTCAATGTGCGCTAACGAGGAAAG	
BamH1-IDR^{FUS} FREE1-F	GGCCTGGACAGCACCGGATCCGCCTCAAACGATTATACCCAAC	*pFREE1::GFP-IDR^{FUS} FREE1* transgenic construct
BamH1-FREE1$^{\Delta IDR}$-F	GGCCTGGACAGCACCGGATCCTCAGTGAAATTTGATCAATC	*pFREE1::GFP-FREE1*$^{\Delta IDR}$ transgenic construct
BamH1-PTAP-IDR^{FUS}	GGCCTGGACAGCACCGGATCCCCTTCTCCGCCGGCGCCG	*pFREE1::GFP-PTAP-IDR^{FUS}FREE1*

FREE1-F		transgenic construct
BamH1-IDR^{FUSm}<br>FREE1-F	GGCCTGGACAGCACCGGATCCGCCTCAA<br>ACGATTCTACCC	*pFREE1::GFP-IDR^{FUSm}FREE1* transgenic construct
BamH1-IDR^{FLOE1}<br>FREE1-F	GGCCTGGACAGCACCGGATCCCACCAAA<br>TAGCCCCACAG	*pFREE1::GFP-IDR^{FLOE1}FREE1* transgenic construct
VPS23A-BD-F<br>VPS23A-BD-R	ATGGCCATGGAGGCCGAATTCGTTCCCCC<br>GCCGTCTAAT<br>CCGCTGCAGGTCGACGGATCCTGAATGTA<br>ACCTACCTGCGAT	VPS23-BD for yeast two hybrid
FREE1-AD-F<br>FREE1-AD-R	GCCATGGAGGCCAGTGAATTCCAACAGG<br>GAGATTACAAT<br>CAGCTCGAGCTCGATGGATCCATGTGCGC<br>TAACGAGGAA	FREE1-AD for yeast two hybrid
FREE1^{ΔIDR}-AD-F	GCCATGGAGGCCAGTGAATTCTCAGTGAA<br>ATTTGATCAATC	FREE1^{ΔIDR}-AD for yeast two hybrid
IDR^{FUS}FREE1-AD-F	GCCATGGAGGCCAGTGAATTCGCCTCAAA<br>CGATTATACCCAA	IDR^{FUS}FREE1-AD for yeast two hybrid
PTAP-IDR^{FUS}FREE1-AD-F	GCCATGGAGGCCAGTGAATTCCCTTCTCC<br>GCCGGCGCCGGCAACC	PTAP-IDR^{FUS}FREE1-AD for yeast two hybrid
FREE1-geno-P1F(P1)<br>FREE1-geno-P2F(P2)<br>FREE1-geno-P3R(P3)	TCGTCATCGTTTCCAATTTCCG<br>TCCGTTCCGTTTTCGTTTTTTAC<br>CTGCTAGCACAAAACCAATTCAC	for genotyping
VPS2-AK71-F<br>GABI_8474	GAAATCTACACAAGCGATGG<br>ATAACGCTGCGGACATCTACA	for genotyping

VPS2-AK2-R	TCACATTTTTCTAAGGTTAT	
PBI221-GFP-F	GGGGACTCTAGAGGATCCATGAGTAAAGG AGAAGAAC	pBI221-FREE1 for protoplast transient expression
PBI221-GFP-R	GATCGGGGAAATTCGAGCTCTCAATGTGC GCTAACGAGG	
PBI221-FREE1-F	GTTCAGGAGGTGGCGGATCCATGCAACA GGGAGATTAC	
PBI221-FREE1-R	GGAAATTCGAGCTCGAATTCTCAATGTGC GCTAACGAGG	
PBI221-FREE1$^{\Delta IDR}$-F	GTTCAGGAGGTGGCGGATCCATGTCAGT GAAATTTGATCAATC	pBI221- FREE1$^{\Delta IDR}$ for protoplast transient expression
PBI221-IDR^{FUS}FREE1-F	GTTCAGGAGGTGGCGGATCCATGGCCTCA AACGATTATACCC	pBI221-IDR^{FUS}FREE1 for protoplast transient expression
PBI221-RHA1-F	GACGAGCTGTACAAGCCCGGGATGGCTAG CTCTGGAAACAAGAAC	pBI221-RHA1 for protoplast transient expression
PBI221-RHA1-R	CGATCGGGGAAATTCGAGCTCAGCACAAC ACGATGAACTCAC	
CHMP 2A shRNA-F	GAAGATGAAGAGGAGAGTGAT	for lentiviral transduction
CHMP 2A shRNA-R	CATGAACAGACAGCTGAAGTT	
CHMP 2B shRNA-F	GCAGCTTTAGAGAAACAAGAA	
CHMP 2B shRNA-R	GCTTGACACCTGCCTTAAATA	
Control shRNA	CAACAAGATGAAGAGCACCAA	
ACTB-F	CATGTACGTTGCTATCCAGGC	for qPCR
ACTB-R	CTCCTTAATGTCACGCACGAT	
CHMP2A-F	CGCGAGCGACAGAAACTAGAG	
CHMP2A-R	CCCGCATCAATACAAACTTGC	
CHMP2B-F	ACGGAAACAGAAGACGAGAAC	

CHMP2B-R	TGCTGTAGTAGACATTGCTCCA	
FUGW-GFR-F	GCAGGTCGACTCTAGAGATGAGTAAAGGA GAAGAACTTTTCAC	for cell transfection
FUGW-GFR-R	GCTTGATATCGAATTTGTACAGTCAATGTGC GCTAACGAGGA	
AM764	ATCCGTCGAAACTAAGTTCTGG	for Saccharomyces cerevisiae expression
AM765	AAGCTTATCGATACCGTCGACCTCG	
AM766	GGTATCGATAAGCTTTCAATGTGCGCTAAC GAGGAAAG	
AM767	TTAGTTTCGACGGATtctagaggatccATGA	
AM768	ACTTCTGCATGATCTTTCTCCTCATT	
AM769	AGATCATGCAGAAGTGTCACAAAGG	
