# Supplementary material for: Biomolecular condensates mediate bending and scission of endosome membranes
Source: Nature. 2024 Oct 9;634(8036):1204–10. doi: 10.1038/s41586-024-07990-0 (PMC11525194; doi:10.1038/s41586-024-07990-0)
Supplement: Supplementary file 2 — Reporting Summary [file 41586_2024_7990_MOESM2_ESM.pdf]

nature portfolio

Corresponding author(s): Xiaofeng Fang, Roland Knorr

Last updated by author(s): Aug 1, 2024

Reporting Summary

Nature Portfolio wishes to improve the reproducibility of the work that we publish. This form provides structure for consistency and transparency in reporting. For further information on Nature Portfolio policies, see our Editorial Policies and the Editorial Policy Checklist.

Statistics

For all statistical analyses, confirm that the following items are present in the figure legend, table legend, main text, or Methods section.

n/a	Confirmed	
☐	☒	The exact sample size (*n*) for each experimental group/condition, given as a discrete number and unit of measurement
☐	☒	A statement on whether measurements were taken from distinct samples or whether the same sample was measured repeatedly
☐	☒	The statistical test(s) used AND whether they are one- or two-sided<br>*Only common tests should be described solely by name; describe more complex techniques in the Methods section.*
☒	☐	A description of all covariates tested
☒	☐	A description of any assumptions or corrections, such as tests of normality and adjustment for multiple comparisons
☐	☒	A full description of the statistical parameters including central tendency (e.g. means) or other basic estimates (e.g. regression coefficient) AND variation (e.g. standard deviation) or associated estimates of uncertainty (e.g. confidence intervals)
☐	☒	For null hypothesis testing, the test statistic (e.g. *F*, *t*, *r*) with confidence intervals, effect sizes, degrees of freedom and *P* value noted<br>*Give P values as exact values whenever suitable.*
☒	☐	For Bayesian analysis, information on the choice of priors and Markov chain Monte Carlo settings
☒	☐	For hierarchical and complex designs, identification of the appropriate level for tests and full reporting of outcomes
☒	☐	Estimates of effect sizes (e.g. Cohen's *d*, Pearson's *r*), indicating how they were calculated

Our web collection on statistics for biologists contains articles on many of the points above.

Software and code

Policy information about availability of computer code

Data collection	All fluorescence imaging data of plant cells were collected on Zeiss LSM880 confocal microscope and Olympus Fluoview FV-1000 confocal laser microscope; ; The super resolution microscopy data was collected using a Nikon combined confocal A1/SIM/STORM system; Imaging of yeast cells was performed on SpinSR10 spinning disc confocal microscope; Transmission electron microscopy data of plant samples was collected using a 80kV Hitachi H-7650 transmission electron microscope (Hitachi High-Technologies Corporation, Japan); Transmission electron microscopy data of mammalian cells was collected using a HT-7800 120 kV transmission electron microscope (Hitachi High-Technologies); Immuno detection of proteins was performed by chemiluminescence (ChemiDoc, LAS4000).
Data analysis	Data analysis was performed in the Fiji implementation of ImageJ (Version 1.51), Cellsens software (Olympus, Japan, Version 4.2), NIS-Elements (Nikon) software (Version Ar), ZEN black software (version 2.3)

For manuscripts utilizing custom algorithms or software that are central to the research but not yet described in published literature, software must be made available to editors and reviewers. We strongly encourage code deposition in a community repository (e.g. GitHub). See the Nature Portfolio guidelines for submitting code & software for further information.

Data

Policy information about availability of data

All manuscripts must include a data availability statement. This statement should provide the following information, where applicable:

- Accession codes, unique identifiers, or web links for publicly available datasets
- A description of any restrictions on data availability
- For clinical datasets or third party data, please ensure that the statement adheres to our policy

Data availability
All data are available in the main text or the supplementary materials. Full version of all gels and blots are provided in Supplementary Figure 1. Source data are provided with this paper. Figures describing the results of the model can be reconstructed directly from the equations and procedures presented in the paper and in the Supplementary Methods, and require no data.

Code availability
The numerical simulation code is available at [cite: https://doi.org/10.5281/zenodo.11919014]. Installation requires the finite element library AMDiS, which can be downloaded at [https://gitlab.math.tu-dresden.de/iwr/amdis]

Research involving human participants, their data, or biological material

Policy information about studies with human participants or human data. See also policy information about sex, gender (identity/presentation), and sexual orientation and race, ethnicity and racism.

Reporting on sex and gender	N/A
Reporting on race, ethnicity, or other socially relevant groupings	N/A
Population characteristics	N/A
Recruitment	N/A
Ethics oversight	N/A

Note that full information on the approval of the study protocol must also be provided in the manuscript.

Field-specific reporting

Please select the one below that is the best fit for your research. If you are not sure, read the appropriate sections before making your selection.

☒ Life sciences ☐ Behavioural & social sciences ☐ Ecological, evolutionary & environmental sciences

For a reference copy of the document with all sections, see nature.com/documents/nr-reporting-summary-flat.pdf

Life sciences study design

All studies must disclose on these points even when the disclosure is negative.

Sample size	Sample sizes are chosen as widely used in the field, for example, Fang et al., Nature 569: 265-269. Biological and technical replicates were performed as described in the Methods for each experiment and conform to standards in the field. Exact n numbers for each experiment are provided in each figure legend.
Data exclusions	No data was excluded from analysis.
Replication	All attempts at replication were successful. All experiments were repeated on different days at least twice. The sample number and biological replicate number are indicated in the legends.
Randomization	Randomization of samples were performed. Seedlings from different plates were collected.
Blinding	Blinding was not deemed necessary in our study since we made no a prior assumptions on the response of the different samples to the experimental treatment, samples were all treated in parallel and all samples treated were always measured.

Reporting for specific materials, systems and methods

We require information from authors about some types of materials, experimental systems and methods used in many studies. Here, indicate whether each material, system or method listed is relevant to your study. If you are not sure if a list item applies to your research, read the appropriate section before selecting a response.

Materials & experimental systems

n/a	Involved in the study
☐	☒ Antibodies
☐	☒ Eukaryotic cell lines
☒	☐ Palaeontology and archaeology
☒	☐ Animals and other organisms
☒	☐ Clinical data
☒	☐ Dual use research of concern
☐	☒ Plants

Methods

n/a	Involved in the study
☒	☐ ChIP-seq
☒	☐ Flow cytometry
☒	☐ MRI-based neuroimaging

Antibodies

Antibodies used

GFP (Roche, 11814460001; 1:7000), FLAG (Merck, F1804; 1:2000), tubulin (Sigma, T5168; 1:2000), ubiquitin (Santa Cruz Biotechnology, sc-8017; 1:1000), His-tag (Sangon, D110002; 1:1000), the horseradish peroxidase (HRP)-conjugated secondary antibodies Goat anti-Mouse (CWBIO, CW0102; 1:10000) and Goat anti-Rabbit (CWBIO, CW0103; 1:10000).

Validation

GFP (Roche, 11814460001): https://elabdoc-prod.roche.com/LifeScience/Document/27e718d4-c7c2-e711-b48d-00215a9b3428
FLAG (Merck, F1804): https://www.sigmaaldrich.com/certificates/sapfs/PROD/sap/certificate_pdfs/COFA/Q14/F3165-1MG0000350182.pdf
tubulin (Sigma, T5168): https://www.sigmaaldrich.com/certificates/sapfs/PROD/sap/certificate_pdfs/COFA/Q14/T5168-100UL0000359065.pdf
His-tag (Sangon, D110002): https://store.sangon.com/productImage/DOC/D110002/D110002_EN_D.pdf
ubiquitin (Santa Cruz Biotechnology, sc-8017; 1:1000): https://datasheets.scbt.com/sc-8017.pdf

Eukaryotic cell lines

Policy information about cell lines and Sex and Gender in Research

Cell line source(s)

COS-7 cells are kind gift from Dr. Yiguo wang (Tsinghua University); SEY6210 WT Saccharomyces cerevisiae cells: Robinson et al. (1988) Mol Cell Biol 8(11):4936-48

Authentication

None of the cell line used have been authenticated.

Mycoplasma contamination

Cells tested negative for mycoplasma contamination.

Commonly misidentified lines
(See ICLAC register)

No commonly misidentified cell lines were used.

Plants

Seed stocks

The free1 mutant (a transposon insertion Arabidopsis mutant line 15-1960-1 from RIKEN) seeds and UBQ10::GFP-FREE1 transgenic seeds were kindly provided by Prof. Liwen Jiang (The Chinese University of Hong Kong, China); The vps2 mutant (a T-DNA insertion line GABI_670D06) seeds were kindly provided by Prof. Youfa Cheng (Chinese Academy of Sciences, China)

Novel plant genotypes

All other transgenic A. thaliana lines (FREE1 and FREE1 variants, FREE1ΔIDR, IDRFUS-FREE1, PTAP-IDRFUS-FREE1, IDRFUSm-FREE1 and IDRFLOE1-FREE1) were generated by Agrobacterium transformation of corresponding constructs into the heterozygous free1 mutant background and subsequent genotyping of homozygous free1 from progeny.

Authentication

Primers used for genotyping T-DNA insertion lines are provided in Supplementary Table 1 in the methods section.
