# Supplementary material for: Biomolecular condensates mediate bending and scission of endosome membranes
Source: Nature. 2024 Oct 9;634(8036):1204–10. doi: 10.1038/s41586-024-07990-0 (PMC11525194; doi:10.1038/s41586-024-07990-0)

Supplementary Figure 1 - Uncropped scans with size marker indications

Ext Data Figure 1b

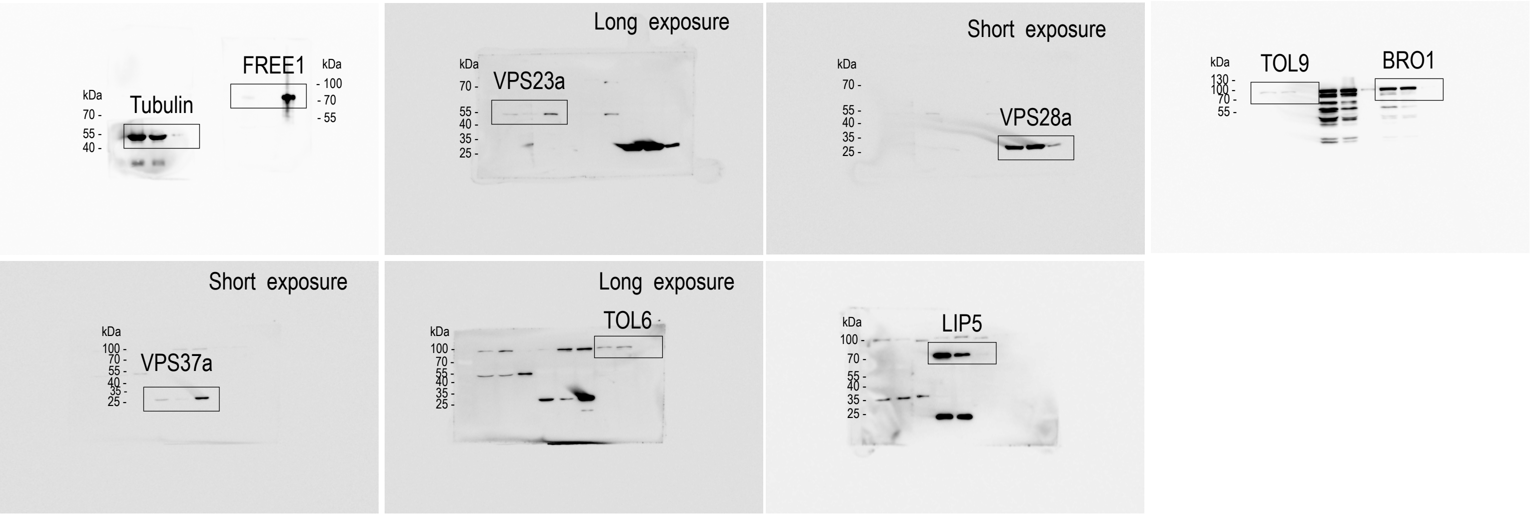

Figure 2h

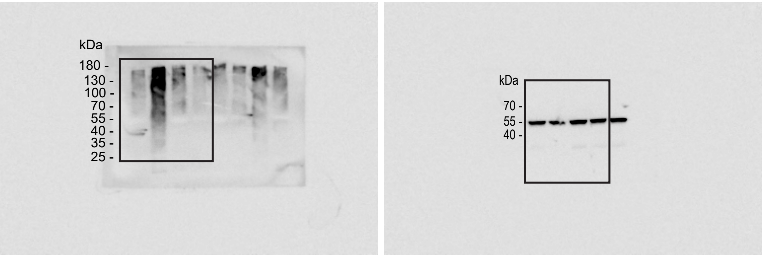

Ext Data Figure 3b

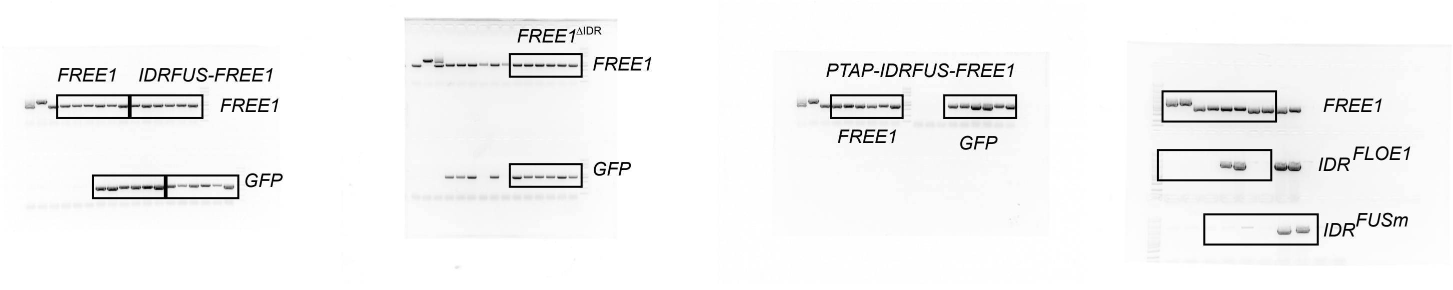

Ext Data Figure 3f

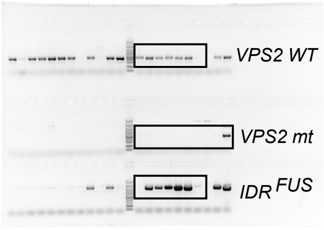

Ext Data Figure 10a

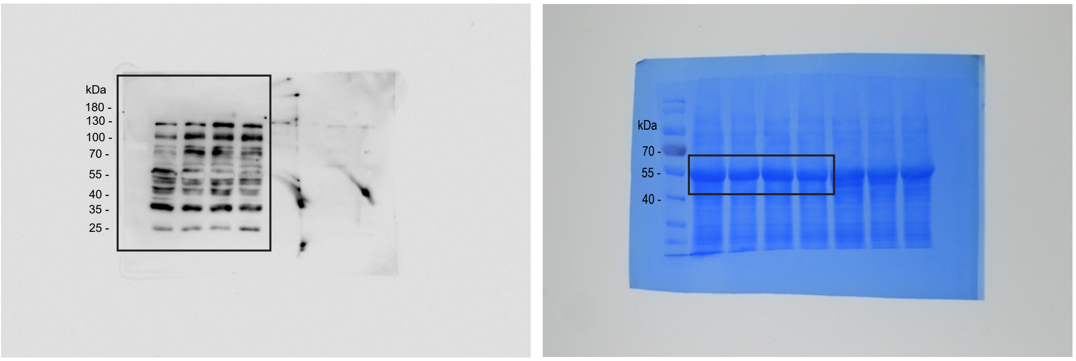

Supplementary Figure 1 - Uncropped scans with size marker indications

Ext Data Figure 4c

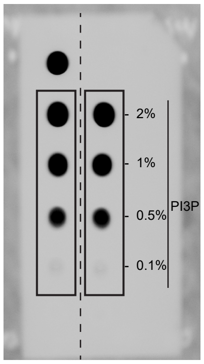

Ext Data Figure 4d

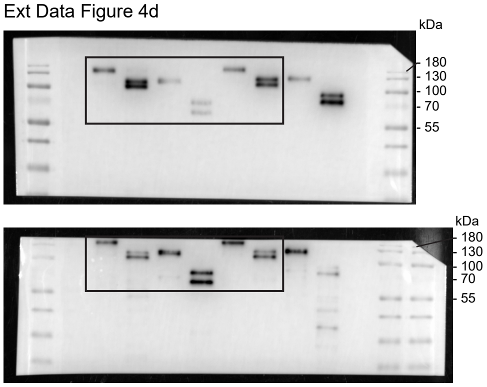

Ext Data Figure 4g

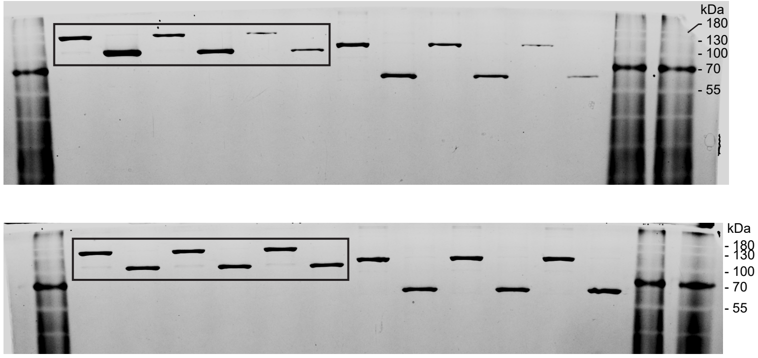

Ext Data Figure 6a

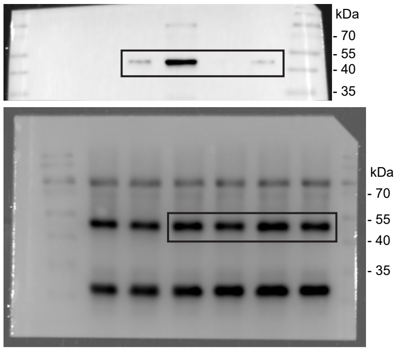

Supplement: Supplementary file 3 — Uncropped gels and blots. [file 41586_2024_7990_MOESM3_ESM.pdf]
